# Investigation of Microplastics (≥10 μm) in Meconium by Fourier Transform Infrared Microspectroscopy

**DOI:** 10.3390/toxics11040310

**Published:** 2023-03-27

**Authors:** Zhiming Li, Jiamin Wang, Xia Gao, Jiaxin Du, Haixia Sui, Jieling Wu, Yizhou Zhong, Boxuan Liang, Yuji Huang, Rongyi Ye, Yanhong Deng, Xingfen Yang, Zhenlie Huang

**Affiliations:** 1 NMPA Key Laboratory for Safety Evaluation of Cosmetics, Guangdong Provincial Key Laboratory of Tropical Disease Research, Department of Toxicology, School of Public Health, Southern Medical University, Guangzhou 510515, China; 2 Beijing Key Laboratory of Organic Materials Testing Technology & Quality Evaluation, Institute of Analysis and Testing, Beijing Academy of Science and Technology (Beijing Center for Physical and Chemical Analysis), Beijing 100089, China; 3 Division III of Risk Assessment, China National Center for Food Safety Risk Assessment, Beijing 100022, China; 4 Department of Healthcare, Guangdong Women and Children Hospital, Guangzhou 511442, China

**Keywords:** microplastics, nanoplastics, meconium, Fourier transform infrared microspectroscopy, risk assessment

## Abstract

Microplastics are prevalent emerging pollutants with widespread distribution in air, land and water. They have been detected in human stool, blood, lungs, and placentas. However, human fetal microplastic exposure remains largely under-studied. To assess fetal microplastic exposure, we investigated microplastics using 16 meconium samples. We used hydrogen peroxide (H_2_O_2_), nitric acid (HNO_3_) and a combination of Fenton’s reagent and HNO_3_ pretreatment methods respectively to digest the meconium sample. We analyzed 16 pretreated meconium samples with an ultra-depth three-dimensional microscope and Fourier transform infrared microspectroscopy. The result showed that H_2_O_2_, HNO_3_ and Fenton’s reagent combined with HNO_3_ pretreatment methods could not digest our meconium samples completely. Alternatively, we developed a novel approach with high digestion efficiency using petroleum ether and alcohol (4:1, *v*/*v*), HNO_3_ and H_2_O_2_. This pretreatment method had good recovery and non-destructive advantages. We found no microplastics (≥10 μm) in our meconium samples, indicating that microplastic pollution levels in the fetal living environment are miniscule. Different results between previous studies’ and ours underscore that comprehensive and strict quality control are necessary for further studies on microplastic exposure using human bio-samples.

## 1. Introduction

Plastics are mass-produced and widely used due to their positive properties and convenience [1]. With imperfect waste plastic management, plastic pollution is inevitable [2]. It has been estimated that plastic waste generation in 2010 was 274 metric tons (Mt), and by the end of 2015, all plastic waste ever generated from primary plastics had surpassed 5800 Mt [3]. Over time, plastics can be broken down into fragments as they undergo various physicochemical processes [4]. Microplastics (MPs, diameter <  5  mm) and nanoplastics (NPs, diameter <  100 nm) are prevailing fragments [5]. In addition to the secondary MPs generated by cracking large mass plastics by force, primary MPs are manufactured to improve the quality of products such as toothpaste and cosmetics [6]. Not only have MPs been detected in aqueous and terrestrial environments, as well as atmospheric ecosystems, but they have also been discovered in human stool [7], blood [8], lung tissue [9], and placenta [10,11]. Several experimental studies have explored the relationship between MP exposure and adverse health effects [12,13,14,15,16]. Nasal exposure to MPs causes MP deposition in the airway and induces an increase in pulmonary inflammatory cells in mice [14]. What is worse, a growing body of evidence indicates that maternal prenatal exposure to MPs increases the risk of progeny injury in mice [12,13,15,16].

Exposure to environmental pollutants in utero is especially concerning because the fetal immune and metabolic systems are still developing, and thus, are more vulnerable to the adverse effects of MPs [17]. The placenta usually protects the fetus by forming a barrier between the matrix and the fetus [18]. A recent study has unveiled an association between human MP exposure and progeny injury [10]. Despite this initial attempt, we cannot determine whether it is directly caused by fetal MP exposure, or indirectly caused by placental MP exposure. Meconium appears in utero around the 13th week of gestation and accumulates thereafter. It is the most common biological sample for in utero drug exposure assessment due to its wide detection window [19]. The composition of meconium contains mucopolysaccharides, water, bile, salts, bile acids, epithelial cells, and other lipids. Meconium is usually excreted in the first 72 h after birth, ranging in quantity from 20 to 70 g [19,20]. Up to 80% of the meconium accumulates after 38 weeks of pregnancy [19,21]. Thus, analysis of meconium provides a direct overview of the fetal exposure, primarily during the last trimester of pregnancy [22]. Due to these advantages, meconium has been used to investigate titanium dioxide nanoparticles’ materno-fetal transfer [23]. Recent studies have shown MPs in placentas and meconium samples [10,11,24,25], and support the link between high MP concentrations and microbiota genera [26]. However, there have been suspicions of contaminated meconium samples in these studies [24,25]. Moreover, they have used limited sample sizes of 2 meconium samples [24] and 4 placental samples [11]. Therefore, the assessment of fetal MP exposure in humans is a crucial unmet challenge, particularly regarding stricter quality control methods and sample sizes [27,28].

In the present study, we established an effective pretreatment method, being suitable to the MP study on meconium. To assess fetal MP exposure, we collected a total of 37 meconium samples and investigated any MPs with diameter > 10 μm in 16 meconium samples using an ultra-depth three-dimensional microscope and Fourier transform infrared microspectroscopy (micro-FTIR). This study finally provides new insight into fetal MP exposure.

## 2. Materials and Methods

### 2.1. Chemicals

Chemicals were purchased from Thermo Fisher Scientific (Waltham, MA, USA), YONGDA (Tianjin, China), Sinopharm Chemical Reagent (Shanghai, China), unless otherwise indicated. All reagents were of analytical grade or higher.

### 2.2. Sample Collection

**Figure 1:**
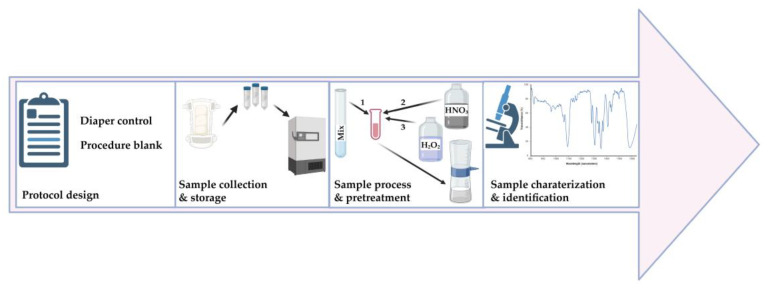
Study overview. Mix: Petroleum ether and alcohol (4:1, *v*/*v*), HNO_3_: Nitric acid, H_2_O_2_: Hydrogen peroxide. The numbers 1–3 represent the order in which the reagents were added.

The Guangdong Women and Children Hospital Scientific Research Committee on Ethics in the Care and Use of Human Samples has approved this study’s protocol (Permit No. 201801057). We collected meconium samples from 37 newborns in November 2021 at the Guangdong Maternal and Child Health Hospital. We obtained the meconium samples by scraping the top portion of meconium from cloth diapers using sterile fecal collectors. We also collected diaper controls for each meconium sample from clean parts of the same diaper. An overview of our study is shown in Figure 1.

### 2.3. Pretreating the Meconium Samples

We initially pretreated meconium samples using hydrogen peroxide (H_2_O_2_), nitric acid (65%, HNO_3_) and a combination of Fenton’s reagent and HNO_3_, which have been applied in previous studies on feces of various species and of human meconium [7,24,25,29]. However, these methods were not suitable for meconium samples in our study. The information of these protocols and samples can be found in the supplementary Table S1. The detailed findings are available in the corresponding Results section in the present study. Thus, we established an effective pretreatment method for meconium. In our experiment, we used ultra-pure water (resistivity ≥ 18.2 MΩ·cm) throughout. First, the samples were freeze-dried under a vacuum at −53 °C. Then, we weighed the samples (0.4038–4.6835 g) and loaded them into prewashed 50 mL transparent glass tubes. After mashing them with glass rods, we added a solution composed of petroleum ether and alcohol (4:1, *v*/*v*) to each tube, and sonicated them repeatedly. After that, the samples were allowed to stand still until they became layered. Then, we discarded the supernatant, added new solution, and sonicated them. We repeated the supernatant exchange operation until the solution became colorless. Next, we discharged the colorless supernatant and completely dried the substrates under nitrogen gas blow. We added HNO_3_ (65%, 5 mL/g meconium) to the substrates and left them overnight in cold water bath. Then, we used a digestion furnace to digest the dissolved substrates for 4 h at 80 °C. For muddy liquid, we added 2 mL HNO_3_ each time and prolonged the digestion time for 30 min until a clear transparent solution was produced, followed by the addition of 5 mL of 30% H_2_O_2_ for 30 min at 80 °C to produce a clear colorless and transparent solution. Next, we filtered the digestion solution with a stainless-steel filter membrane (10 μm) and a suction device. To remove any remaining grease and promote the MP recovery rate, we rinsed the filters and containers several times in 70 °C water. Finally, we used toothless stainless-steel tweezers to remove the filter membranes, placed them into petri dishes, and either air-dried or dried them with a 50 °C heating element. We set three procedure blank controls (without meconium) and pretreated them in the same way. Finally, we only pretreated 16 meconium samples from 37 newborns due to the limited quality (sample weight was small) of the meconium sample from 21 newborns to assess fetal MP exposure.

### 2.4. Recovery Experiment

We performed the recovery experiment using 50 μm diameter and 200 μm diameter polystyrene (PS) standard before sample detection. We added PS standard to 10 mL HNO_3_, then left it at room temperature overnight. Then, we conducted digestion at 80 °C for 4 h. We added 2 mL H_2_O_2_ to the solution and conducted digestion again at 80 °C for 1 h. Then, we added 70 °C ultra-pure water to the digestion solution until there was 50 mL of it and filtered it using a 10 μm stainless-steel filter membrane and a suction device. In addition, we rinsed the container and filtered the membrane several times using 70 °C hot water. Finally, we used toothless stainless-steel tweezers to remove the filter membrane and put it into a petri dish, and either air-dried or dried it with a 50 °C heating element. After treatment, the number of MPs was counted with an ultra-depth three-dimensional microscope and the recovery rates of MPs were calculated. Three replicates were conducted.

### 2.5. Analysis of Samples by Ultra-Depth Three-Dimensional Microscope and Micro-FTIR

We observed the color, size, and shape of the targets (potential MPs) on a stainless-steel membrane with an ultra-depth three-dimensional microscope (VHX-600, Keyence, Osaka, Japan). Before observation, we adjusted the white balance for color correction. Next, we put the filter membrane under the objective lens. Then, we performed micro-FTIR (HYPERION2000, Bruker, Karlsruhe, Germany) to identify the target objects (potential MPs). We analyzed in the attenuated total reflectance mode with a mercury–cadmium–telluride detector, with a wavenumber range of 400–4000 cm^−1^ and a resolution of 4 cm^−1^. We examined the background of the air by aligning the beam with air, scanned the target objects, and collected the infrared characteristic spectra. The collected infrared characteristic spectra had been reduced from the background spectra of the air. We compared the target objects’ infrared spectra with the Omnic and Bruker reference spectra database, and conducted qualitative analysis to determine the suitability of the fit. If the spectral similarity exceeded 70% when compared with the reference MPs, and infrared expert could detect the presence of characteristic infrared peaks, we defined the particles as MPs.

### 2.6. Quality Control

To minimize the risk of MP contamination from other sources, we applied various measures. Our colleagues received training to familiarize them with the experimental process, including meconium collection. We handled the samples in an isolated, clean, and windowless room with restricted access. Throughout every step of the laboratory work, we wore clean cotton laboratory coats and natural latex gloves. Additionally, we rinsed all utensils with ultra-pure water and dried them in an oven before use. We also collected diaper controls to exclude the possible contamination during meconium sampling. The diaper controls were collected by clean scissor (rinsed with prefiltered water) from the surface of cloth diapers into sterile fecal collectors. The details about the diapers can be found in the supplementary Figure S1. At the meanwhile, 3 procedure blank controls were performed to rule out the possibility of contamination from sample pretreatment and the examination process.

## 3. Results

**Figure 2:**
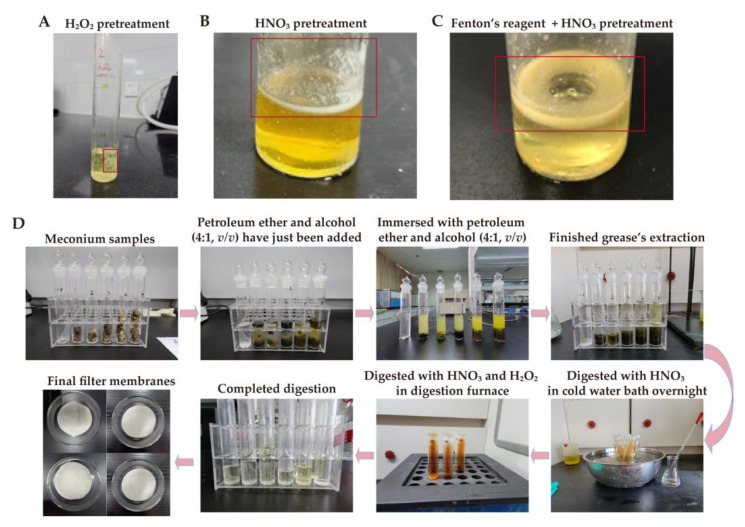
Different effects of various digestion methods. (**A**) After digestion with H_2_O_2_ for nine months; (**B**) After HNO_3_ digestion; (**C**) After digestion by a combination of Fenton’s reagent and HNO_3_; and (**D**) The process of our established pretreatment methods. The red boxes in (**A**–**C**) indicate incomplete digestion.

After H_2_O_2_ pretreatment for nine months, the meconium samples had yet to fully digest, and this affected the MP observation (Figure 2A). In addition, the meconium samples were also not fully digested after treatment with HNO_3_, nor with a combination of Fenton’s reagent and HNO_3_, respectively (Figure 2B,C). However, with our new pretreatment methods, meconium samples could be completely digested (Figure 2D). A comparison of these pretreatment methods is shown in Table 1.

**Figure 3:**
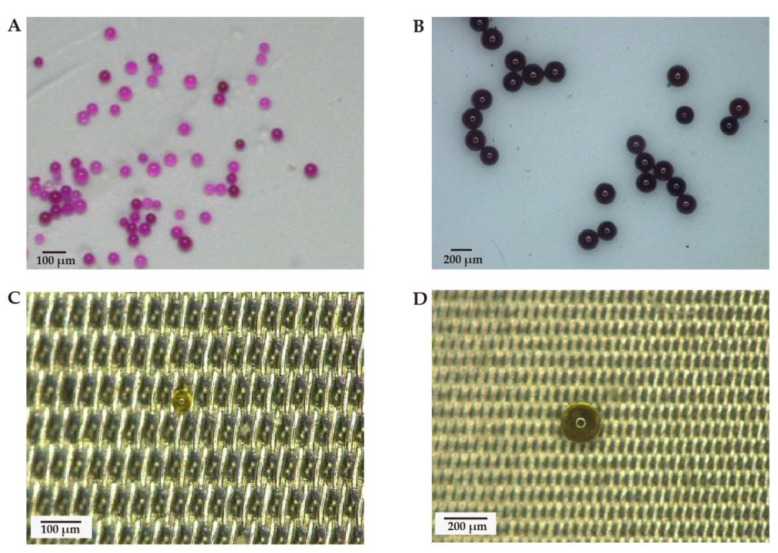
PS standard before/after digestion. (**A**,**B**) Before digestion, 50 μm and 200 μm, respectively; and (**C**,**D**) After digestion, 50 μm and 200 μm, respectively.

In the recovery experiment, 50 μm and 200 μm PS-MP standard particles initially showed a spherical shape (Figure 3A,B). After pretreatment, two types of PS-MP standard particle also appeared as spheres (Figure 3C,D). The average recoveries of 50 μm and 200 μm PS-MP particles were 84.69% and 66.80%, respectively (Table 2). These results suggested that the pretreatment method was feasible.

**Figure 4:**
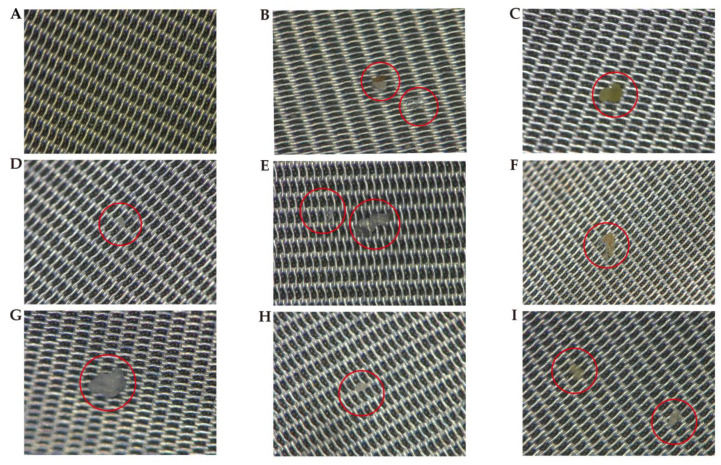
Microscope with field image super depth (200× magnification). (**A**) Procedure control and (**B**–**I**) Representative samples. Red circles mark potential MPs.

**Figure 5:**
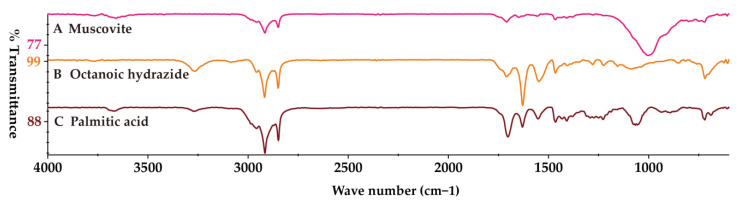
The acquired FTIR spectrum. (**A**) Muscovite, (**B**) Octanoic hydrazide, and (**C**) Palmitic acid.

Then, we identified several potential MPs via an ultra-depth three-dimensional microscope (Figure 4) within 16 meconium samples. Furthermore, we measured all the collected microparticles by micro-FTIR. However, micro-FTIR did not detect MPs in any of the meconium samples, whereas we observed muscovite, octanoic hydrazide, and palmitic acid in some of the meconium samples (Figure 5). Additionally, the FTIR spectra of other potential MPs in the meconium samples could be found in the supplementary Figure S2. However, these FTIR spectra could not be characterized as defined chemical compounds based on spectral similarity when compared with Omnic and Bruker reference spectra databases. Details about the sample and the results are presented in Table 3.

## 4. Discussion

A growing body of literature has revealed that MPs are present in humans. However, human fetal MP exposure remains an under-researched topic. We found no MPs > 10 μm in any of the meconium samples, under strict quality control. This suggests that the MP pollution levels in the fetal living environment were miniscule in at least the samples analyzed.

Most MP exposure has been discovered in marine life [34]. Thus, we speculate that pregnant women living near bodies of water may be more likely to inadvertently ingest MPs via seafood than those people living far from water. Hence, our meconium samples are likely to present MPs due to the fact that the Guangdong Maternal and Child Health Hospital is located near the Pearl River in which MPs have been reported [35,36]. In a recent study, plastic particles in human blood were investigated, and it was estimated that the mean of the sum of the MP concentrations for each donor was 1.6 μg total plastic particles/mL blood sample [8,27,28]. Another study showed that MP fragments with diameter ranging from 5 to 10 μm can reach human placenta tissues at all levels [11]. Therefore, we speculate that MPs enter the placenta via maternal blood because the placenta connects the fetus and matrix via the bloodstream [37]. Maternal blood delivering nutrients to the fetus may also deliver MPs to the fetus, which would be detrimental to health. PS-MPs have caused adverse effects on pregnancy outcomes in mice via immune disturbance [38]. Given that immune disturbance has weakened placenta barrier function [39], MP exposure may weaken placenta barrier function and allow MPs to penetrate it to arrive at fetus. Besides, amniotic fluid is considered a transudate of plasma from the mother across the uterine decidua and/or the placenta surface [40]. The fetus consumes an average of 210–760 mL of amniotic fluid per day [41]. The detection of MPs in the placenta hints that amniotic fluid could be another source of fetal MP exposure. Once MPs intrude the amniotic fluid, they are detectable in meconium. However, to date there has been no report on amniotic fluid MPs. This requires further study and will clarify fetal exposure routes.

Digestion is the first step in separating MPs from environmental and biological samples. Various chemicals have been used for the digestion. For instance, 30% H_2_O_2_ has been used to digest biological tissue and meconium, and the result has shown that 30% H_2_O_2_ at 25 °C takes about 5 weeks to eliminate the organic matter from the meconium samples [24]. However, it failed to digest our meconium samples completely under the same conditions (Figure 2A). In our study, after 30% H_2_O_2_ had digested for nine months, a considerable amount of residue remained in the solution, which affected MP detection. Different sample sources may cause varying H_2_O_2_ digestive capacity in meconium. Meconium consists of water, lipids, protein, sterols, and cholesterol precursors derived from swallowed amniotic fluid, shed epithelial cells, and intestinal secretions [20]. We found that there was a considerable amount of grease after 30% H_2_O_2_ digestion, which could be explained by the wealth of lipids in our meconium samples, and the limited ability of H_2_O_2_ in removing organic matter [42]. HNO_3_ digestion, applied in a recent MP study on meconium [25], also showed limited digestive capacity in our study (Figure 2B). HNO_3_ digestion is an efficient method for removing organic matter [33], whereas it failed to digest our meconium samples completely. This might also have been attributed to the different sample sources. The wealth of lipids in our meconium samples made HNO_3_ difficult to completely digest. Moreover, we attempted to digest meconium using Fenton’s reagent, combined with HNO_3_, which have been used in an MP study on the feces of various species [29]. Fenton’s reagent has shown high digestion efficiencies on organic matter [42]. Nevertheless, the attempt did not succeed in completely digesting our meconium samples. Rather, it behaved as grease residue in the solution and subsequently affected MP separation (Figure 2C). After these trials, we established an effective method for meconium pretreatment. Foremost, we selected a petroleum ether and alcohol (4:1, *v*/*v*) mixed solution at the initial pretreatment step to extract any grease which had previously hindered digestion. After that, we chose HNO_3_ to digest any remaining solid matter. Finally, we added H_2_O_2_ to digest any remaining organic matter and obtained a clear solution for further MP extraction and detection. Our pretreatment methods showed good recovery (Table 2) and no significant changes in the MP properties after the extraction process (Figure 3). This indicated that our methods for extracting MPs from meconium with no damage had been effective. We also compared several attempted methods with our methods in Table 1. In sum, our pretreatment methods were effective and time-saving in the MP meconium study.

In the present study, we did not detect MPs in any of the 16 meconium samples. Conversely, a previous study has identified MPs in two meconium samples [24]. However, the number of samples is a limitation of their generalizability. In our study, we used a similar amount of samples as other well-designed meconium studies [23,43]. A most recent study also identified MPs ranging from 20 to 500 μm in 12 meconium samples (median: 51.4 particles/g) [25]. These results were inconsistent with ours, which may have been due to different sample sources and detection methods. Furthermore, contamination during sample collection and analysis could not be excluded in existing meconium MP studies [24,25], whereas we found no sample contamination in our study. Our pretreatment method showed good recovery but we found no MPs in any of our meconium samples. This result was attributed to several reasons. Firstly, we focused on MPs with diameter >10 μm and the placenta barrier prevents most of these macromolecular substances from entering the fetus [37]. In the most recent study of MPs in the placenta, only 6 MPs were found ranging from 7.3 to 27.6 μm in 4 out of 30 participants in normal pregnancies [10]. Furthermore, a recent study reported only 12 MPs in 4 human placentas [11]. These results indicated that human placental MP exposure levels are miniscule. Therefore, low MP concentrations could be the major reason for our result.

The limit of FTIR could be another reason. FTIR has been used to detect contaminants in many environmental samples [44,45,46]. It also has been used to detect MPs from stool [47]. However, FTIR can only accurately detect irregular particles with diameter > 50 μm [48]. It is unable to detect those irregular MPs with diameter ranging from 10–50 μm in the meconium samples. A recent study demonstrates that the majority of detected MPs (64%) are smaller than 10 μm, and the shape of those ranging from 10 to 34.5 μm is irregular (fragment, film, pellet, fiber) in the placenta [10]. A previous study also revealed that MPs with diameter < 10 μm were able to penetrate the placenta because MPs with diameter ranging 5–10 μm were detected in the placenta [11]. These results suggest that most MPs in the human placenta are smaller than 10 μm, and as for MPs ranging from 10–50 μm in the placenta, they all have irregular shapes. Even though two studies have shown MPs with diameter > 50 μm in the placenta and in the meconium [24,25], the authors suspect that the negative controls may have been contaminated by MPs, thus weakening the results’ credibility. PS beads with a diameter up to 240 nm are taken up by the placenta and can penetrate the placental barrier in ex vivo dual recirculating human placental perfusion model [49]. Several studies have also confirmed that nano-sized MPs can infiltrate the fetus [12,15,50]. Besides, nano-sized particles could pass through the human placenta barrier and appear in meconium. For example, transmission electron microscope analysis has shown TiO_2_ particles ranging from 10 to 225 nm in diameter in meconium [23]. In sum, we found no MPs in meconium, and this may indicate low-level MPs, irregularly shaped MPs ranging from 10–50 μm, or MPs with diameter < 10 μm in meconium.

Although we observed no MPs in meconium, we found muscovite, octanoic hydrazide, and palmitic acid in the meconium samples (Figure 5). Muscovite, also called white mica, is a major component of traditional Chinese medicine, which has been utilized to treat bleeding, dysentery, and inflammation [51]. Muscovite has been developed and marketed in China for the treatment of gastric disease [52,53]. Considering that women often use traditional Chinese medicine during pregnancy [54], it was not surprising that we observed muscovite in the meconium. This reminded us of the potential for inorganic particle exposure in human fetuses. Palmitic acid is the most abundant saturated fatty acid in the blood [55], and its concentration in amniotic fluid is considered the most reliable method for assessing fetal lung maturity [56]. The presence of palmitic acid in meconium indicated that meconium’s grease had not been eliminated. In order to remove grease in the meconium samples in our study protocol, we used a mixed solution composed of petroleum ether and alcohol (*v*/*v*, 4:1). Additionally, we used hot water to dissolve as much grease as possible. Indeed, we only found palmitic acid in one meconium sample, and the presence of palmitic acid did not affect MP detection. The present study has several limitations. Firstly, we did not make the connection between our results and the mothers’ exposure. Secondly, in our study, we only focused on MPs with diameter > 10 μm. However, nano-sized MPs deserve attention because they are more prone to penetrating the human body due to their small diameter. To detect nano-sized MPs, further study should employ higher spatial resolution instruments such as surface-enhanced Raman spectroscopy, mass spectrometry, and combinations of various detection methods [57]. Thirdly, we selected PS standard as representative MPs in the recovery experiment, similar to how PS has been found in existing meconium MP studies [24,25]. It should also be mentioned that MPs’ response to the digestion method varies with different materials [32]. Lastly, although our pretreatment methods showed good recovery, MPs in meconium may be lost during sample preparation due to the extraction conditions (HNO_3_ and heating to 80 °C) [58]. Increasing evidence of MPs in human bio-samples has aroused public attention and concern. This has alerted the scientific community of MP exposure-risk in humans, as well as the need to promote plastic management.

## 5. Conclusions

We developed an effective pretreatment method to separate microplastics from meconium. Using our new pretreatment method, we detected no MPs (>10 μm) in meconium by comparing with Omnic and Bruker reference spectra databases. This suggested low fetal MP exposure levels in newborns. Further studies should focus on fetal nanoplastics exposure. In addition, the existence of NPs in the fetal body and their related metabolic pathway also warrants further study. However, we should not overlook the limitations of the human data presented in the previous studies when we conduct risk assessment on human microplastics exposure.

## Figures and Tables

**Table 1:** The comparison of different chemical digestion methods.

Chemical	Hydrogen Peroxide (H_2_O_2_)	Nitric Acid (HNO_3_)	Fenton’s Reagent + HNO_3_	Petroleum Ether and Alcohol + HNO_3_ + H_2_O_2_
Sample	Feces [7,30,31] Meconium [24]	Meconium [25] Oyster [32] and clam tissue [33]	Feces [29]	Meconium Present study
Method summary	25 mL 30% H_2_O_2_ with 3 g human fecal samples for 20 days [7]. 30% H_2_O_2_ at 25 °C for two weeks [30]. 30% H_2_O_2_ and samples were mixed in a 1:1 *v*/*v* ratio, incubated in a sand bath (~75 °C) for 24 h and then at room temperature for 36 to 48 h [31]. H_2_O_2_ for 5 weeks to completely eliminate the organic matter [24].	HNO_3_ is added to the samples, allowing to stand for 48 h, and then heat at 95 °C for at least 3 h [25]. HNO_3_ is added to the samples and incubate at 60 °C for 24 h [32]. Clam tissue digested in 40 mL of 69–71% HNO_3_ for 4 h in a hot water bath (~90 °C) [33].	Phase 1: 140–700 mL Fenton’s reagent (H_2_O_2:_ iron catalyst solution = 2.5:1) with human, chicken, and zebrafish feces, lasting less than 5 h below 40 °C. Phase 2: 65% HNO_3_ is added and incubated in 50 °C water bath for 30 min.	Phase 1: Petroleum ether and alcohol (4:1, *v*/*v*) remove lipids. Phase 2: HNO_3_ (5 mL/g meconium) is added and incubated in cold water bath overnight, followed by digestion for 4 h at 80 °C. Phase 3: 5 mL H_2_O_2_ is added for 30 min at 80 °C, followed by filtering the digestion solution.
Advantage	Easy to operate; extract MPs with no damage.	Efficient and feasible way for meconium digestion; time-saving.	Easy to operate; time-saving	Completely digest our meconium samples; easy to operate; time-saving.
Disadvantage	Could not completely digest our meconium samples; time-consuming	Could not completely digested our meconium samples; potentially damage MPs.	Could not completely digest our meconium samples.	Potentially damage MPs

**Table 2:** The recovery rate of 50 μm and 200 μm PS standard in the test.

Group	Added Targets/Item		Recovery Targets/Item		Recovery Rate (%)	
	50 μm	200 μm	50 μm	200 μm	50 μm	200 μm
1	293	87	258	65	88.05	74.71
2	255	63	181	31	70.98	49.21
3	726	34	690	26	95.04	76.47
Average recovery rate (%)	-	-	-	-	84.69 ± 12.38	66.80 ± 15.26

“-“: Not available.

**Table 3:** Summary of sample information and results of MP detection.

Sample Description	Dry Weight/g	Results
Procedure blank control 1	-	Negative
Procedure blank control 2	-	Negative
Procedure blank control 3	-	Negative
Meconium No. 1	1.8600	Negative
Meconium No. 2	0.8013	Negative
Meconium No. 3	2.1695	Negative
Meconium No. 9	2.5295	Negative
Meconium No. 10	2.8588	Negative
Meconium No. 12	4.3440	Negative
Meconium No. 14	3.0063	Negative
Meconium No. 15	0.4038	Negative
Meconium No. 16	0.9895	Negative
Meconium No. 17	4.6835	Negative
Meconium No. 18	1.4887	Negative
Meconium No. 19	1.4463	Negative
Meconium No. 29	2.1207	Negative
Meconium No. 31	1.7712	Negative
Meconium No. 32	0.7137	Negative
Meconium No. 37	2.1840	Negative

“-“: Not available.

## Data Availability

The data presented in this study are available on request from the corresponding author.

## Supplementary Materials

The following supporting information can be downloaded at: https://www.mdpi.com/article/10.3390/toxics11040310/s1, Table S1: The information of protocols and samples used in the initial pretreatments; Figure S1: The composition of diaper control. (**A**) Photograph of Fourier transform infrared microspectroscopy (200 × magnification); (**B**) FTIR spectrum of diaper. The red dot in A indicates the position for detected; Figure S2: FTIR spectra of other potential MPs in the meconium samples from (**A**) No. 1, (**B**) No. 9, (**C**) No.10, (**D**) No. 15, (E) No. 17, (**F**,**G**) No. 32, (**H**) No. 37.
