# Supplementary material for: Investigation of Microplastics (≥10 μm) in Meconium by Fourier Transform Infrared Microspectroscopy
Source: Toxics. 2023 Mar 27;11(4):310. doi: 10.3390/toxics11040310 (PMC10143218; doi:10.3390/toxics11040310)
Supplement: Supplementary file 1 [file toxics-11-00310-s001.zip › toxics-2288274-supplementary.pdf]

Supporting Information

Investigation of Microplastics (≥10 μm) in Meconium by Fourier Transform Infrared Microspectroscopy

Zhiming Li [1,†], Jiamin Wang [2,†], Xia Gao [2], Jiaxin Du [1], Haixia Sui [3], Jieling Wu [4], Yizhou Zhong [1], Boxuan Liang [1], Yuji Huang [1], Rongyi Ye [1], Yanhong Deng [1], Xingfen Yang [1] and Zhenlie Huang [1,*]

[1] NMPA Key Laboratory for Safety Evaluation of Cosmetics, Guangdong Provincial Key Laboratory of Tropical Disease Research, Department of Toxicology, School of Public Health, Southern Medical University, Guangzhou 510515, China
[2] Beijing Key Laboratory of Organic Materials Testing Technology & Quality Evaluation, Institute of Analysis and Testing, Beijing Academy of Science and Technology (Beijing Center for Physical and Chemical Analysis), Beijing 100089, China
[3] Division III of Risk Assessment, China National Center for Food Safety Risk Assessment, Beijing 100022, China
[4] Department of Healthcare, Guangdong Women and Children Hospital, Guangzhou 511442, China
* Correspondence: huangzhenlie858252@smu.edu.cn; Tel.: +86-20-61648415
† These authors contributed equally to this work.

Prepared for ***Toxics***

This Supporting Information contains 2 figures and 1 table.

Table S1. The information of protocols and samples used in the initial pretreatments.

Chemical	Protocol	Sample	Dry weight/g
Fenton's reagent + Nitric acid (HNO_3)	Phase 1: 280 mL Fenton's reagent (H_2O_2: iron catalyst solution = 2.5:1) with meconium, lasting less for 5 h below 40 °C.<br>Phase 2: 4 mL 65% HNO_3 is added and incubated in 90 °C water bath for 5 h.	1A<br>2A<br>3A	1.23<br>1.35<br>1.23
Hydrogen peroxide （H_2O_2）	30% H_2O_2 with human fecal samples (25 mL:3 g) for 9 months.	4A<br>5A<br>6A	0.33<br>0.20<br>0.26
HNO_3	HNO_3 (9 mL) is added to the samples (0.1 - 0.2 g), allowing to stand for 16 h, and then heat at 80 °C for 4 h.	7A<br>8A<br>9A<br>10A<br>11A	0.31<br>0.20<br>0.21<br>0.19<br>0.18

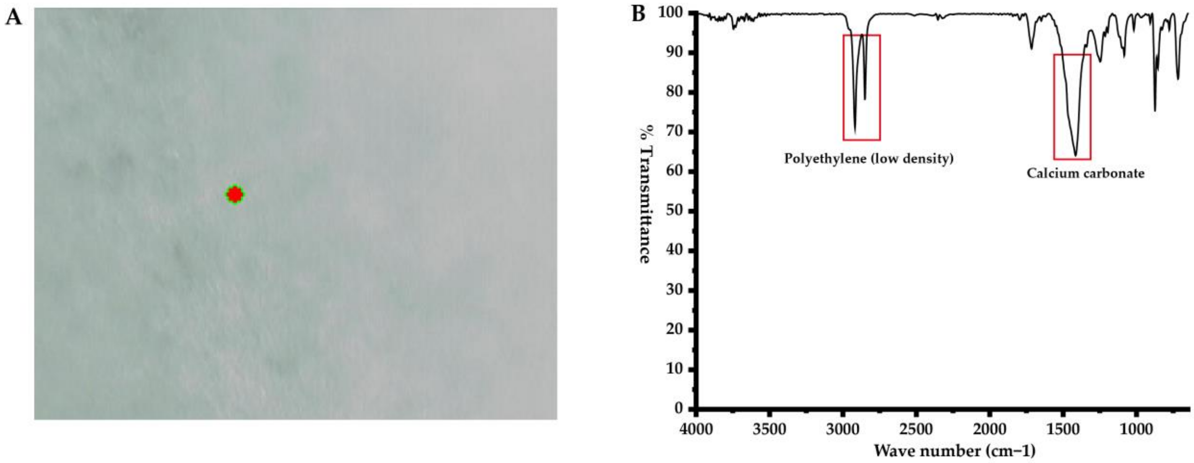

Figure S1. The composition of diaper control. (**A**) Photograph of Fourier transform infrared microspectroscopy (200 × magnification); (**B**) FTIR spectrum of diaper. The red dot in A indicates the position for detected.

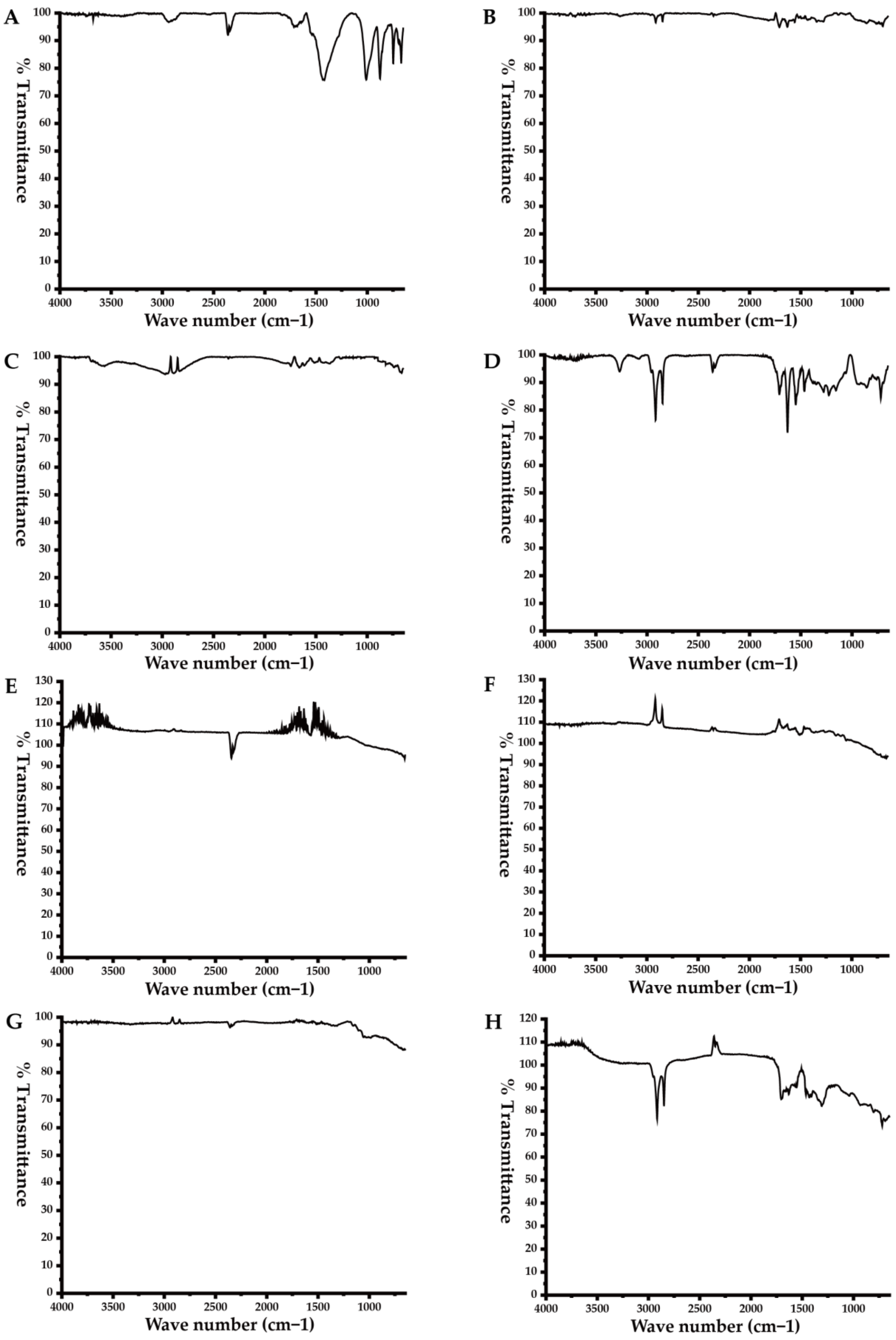

Figure S2. FTIR spectra of other potential MPs in the meconium samples from (**A**) No. 1, (**B**) No. 9, (**C**) No.10, (**D**) No. 15, (**E**) No. 17, (**F**,**G**) No. 32, (**H**) No. 37.
